# The social burden experienced by families caring for members living with cancer in KwaZulu-Natal, South Africa

**DOI:** 10.4102/phcfm.v13i1.2955

**Published:** 2021-10-25

**Authors:** Phindile C. Mlaba, Themba G. Ginindza, Khumbulani W. Hlongwana

**Affiliations:** 1 Department of Public Health Medicine, Faculty of Health Sciences, University of KwaZulu-Natal, Durban, South Africa

**Keywords:** cancer, family caregiver, social burden, support, experience, KwaZulu-Natal

## Abstract

**Background:**

Cancer is a global public health problem and it affects people in different ways. Family caregivers (FCs) play an essential role in caring for patients with cancer, and thus, they experience many caregiver burdens that go unnoticed.

**Aim:**

This research study explored the social burden that families experience in providing care to their family members living with cancer.

**Setting:**

This study was conducted in Durban and Pietermaritzburg, cities located in KwaZulu-Natal, South Africa.

**Methods:**

This was a qualitative study using the interpretative phenomenological approach that was ideal for understanding FCs subjective perspectives on their cancer caregiving experience. Data saturation were reached at 20 in-depth interviews.

**Results:**

Two major themes culminated from the data analysis; dynamics of a cancer diagnosis and psychosocial impact of a cancer diagnosis with respective sub-themes. Themes centred around the relational impact of a cancer diagnosis with FCs experiencing a shift in this dynamic and a disturbance to normality in social life. Social support systems were found to play a meaningful role in mitigating the impact of a cancer diagnosis with financial, psychosocial and educational support considered essential needs.

**Conclusion:**

Cancer caregiving is a challenging task that also presents opportunities for strengthening family bonds as they evolve in new paths. A family-centred care approach is recommended as a form of social support with further collaboration with health care providers for guided patient care. If the needs of FCs are addressed accordingly through health care policies and interventions, FCs may be able to provide better care and support for their family members with cancer and thus positively impact cancer survivorship.

## Background

Cancer is a public health problem, globally.^1^ It is predicted that sub-Saharan Africa (SSA) will have an increase of more than 85% in cancer burden by 2030.^2^ This suggests that there will also be a substantial increase in families who care for family members living with cancer in SSA.^2^ In 2016, one in four people was estimated to be affected by cancer, annually.^1^ Cancer affects people in different ways, either through being diagnosed with it or by having a family member or close person diagnosed with it.

Evidence has revealed that a cancer diagnosis actually has a greater impact on the family members than on the patients.^3^ Family members often find themselves in compelling situations to take on caregiving responsibilities without an option of considering whether they possess the skills set for such tasks.^4^ It is apparent that family caregivers (FCs) are also in need of care and support for their psychological health, financial and social well-being, as the caregiving burden is frequently a result of an imbalance between these support needs and caregiving demands.^3,5,6^ Family caregivers play an essential role in the care of patients with cancer as they are the most critical source of support to the patients that they care for.^3,5,7^ As such, FCs often face various difficulties within their role and suffer from many unmet needs and caregiver burdens.^6^ Therefore, social support from other family members, friends and the health system is required to lighten the caregiving burden and promote the well-being of FCs.^8^

The caregiving role disrupts the social life of FCs as their time and energy get focused on the patient with cancer and the recovery thereof.^3^ This has been found in global studies addressing this topic where research participants reported having had drastic disruptions to their social lives and experienced a loss of normality in life with cancer caregiving posing a negative effect on their holiday time, travel, hobbies and general socialising.^3,7,9,10^ In a study conducted amongst South African FCs, it was revealed that the daily activities of feeding, cleaning and dressing of the patient were the most challenging and time-consuming part of the caregiving process.^11^ In another study of the experiences of South African FCs, participants reported to have experienced a loss of contact and closeness with others and were unable to continue with planned activities, as a result of the commitment to caregiving.^1^ Although caregiving may result in a loss of contact and closeness with others, further research has reported a positive attribute, whereby a closer relationship and a sense of connectedness with the patient are established, as a result of caregiving.^6^

Research has suggested that a supportive family structure and a three-part collaborative partnership, inclusive of patients, healthcare providers and family members, is likely to lighten the caregiving burden that FCs face in caring for their family members with cancer.^12,13^ Research has also revealed that providing support to the specific needs of FCs could increase the resilience levels to caregiving demands and improve the well-being of FCs.^14^ Evidence further suggests that there is no clear relationship between healthcare professionals and FCs regarding the collaboration of formal and informal healthcare of cancer patients, which can facilitate the support and integration of FCs into cancer care.^15^ It is suggested that policymakers need to recognise the value and importance of FCs, as well as identify their unmet needs concerning the caregiving role.^4^ This study was aimed at exploring the social experiences of the families caring for members living with cancer in KwaZulu-Natal (KZN), South Africa.

## Methods

### Study design

This study was conducted through qualitative methods, as it explored the experiences of FCs caring for their family members with cancer.^16^ It was rooted in the interpretative phenomenology study design, because of its ability to explore and understand the cancer caregiving experience by interpreting the subjective perspectives of the FCs and their lived experiences of caring for a family member with cancer.^17,18^

### Study setting

This was a community-based study and was conducted in the province of KZN in South Africa, with the study sites located in Durban and Pietermaritzburg, respectively. The study sites were Chatsworth, uMlazi and Wentworth, which are located in Durban, alongside iMbali and Sobantu located in Pietermaritzburg. The interviews were conducted in the participants’ homes or at a setting preferred by the participant within the respective community.

### Study population, sample size and approach to sampling

The participants in this study were primary FCs residing in KZN who were or had previously been involved in the daily processes of caring for a family member with cancer. To be included in the study, participants had to fall within the eligibility criteria in terms of the target population, age and consent. The data saturation was reached at 20 in-depth interviews bringing the total number of participants to 20.^19^

A purposive sampling method was applied in this study where participants were recruited through referral by patients who attended cancer support groups and non-government organisations (NGOs), such as hospices and homes for the sick in KZN.^20^ The support groups and NGOs were identified in Durban and Pietermaritzburg where the research team attended the support group meetings and met with the leaders of the NGOs to present the research study and then gained access to patients with cancer. The patients then referred the research team to their primary caregivers who were then approached and recruited to participate in the study. Snowball sampling was used to complement the purposive sampling method, an approach that was useful to gaining access to the potential participants who were not accessible via the cancer support groups and NGOs.^16^ This was achieved by consulting with already identified participants who shared about other potential participants whom they knew.^16^ The potential participants were then approached by the research team and recruited to participate in the study. This method was a useful approach to gaining access to the former FCs of the late cancer patients whom they once cared for. To reduce potential sampling bias, all identified potential participants were part of a targeted population of FCs who cared for a family member who had cancer. Sampling was guided by the eligibility criteria of which all potential participants who satisfied the criteria were included in the study.

### Data collection

A semi-structured interview guide was used to conduct the interviews.^21^ The topics covered in the interviews centred around the FC’s family and caring roles, social activities and life in general, as well as the dynamics of these since the cancer diagnosis. The topics included in the interview guide were informed and guided by literature on the psychosocial, economic and family aspects of cancer care. The interviews were conducted at the participants’ homes, with only one participant preferring to be interviewed at a local library in the community. The lead investigator conducted all the interviews, which were audio recorded, with the participants’ permission. Hand-written field notes were also taken during the interviews. Interviews were conducted in the participants’ preferred languages that were either English or isiZulu. The interview guide was prepared in English and IsiZulu of which the lead investigator is fluent in both languages. As a Masters student with shared socio-demographic characteristics with some participants and limited personal experiences with family caregiving, the lead investigator engaged in reflexivity through continuous consultation with supervisors and colleagues regarding the research topic and processes. This promoted the lead investigator’s self-awareness, introspection and neutrality that allowed for pure and uninfluenced participant responses and study outcomes.^22,23^

### Data analysis

Interview recordings were transcribed verbatim and transcripts were translated to English as some of these were in IsiZulu. They were then transferred to the NVIVO 12 software for qualitative data analysis where the data were coded and deductively organised into the respective themes that were developed using the interview guide questions and guided by literature.^24,25^ The six steps of thematic analysis were then applied to identify and report patterns within the data.^26,27^

#### Six-step thematic analysis

**Step 1:** All transcripts were read and re-read thoroughly for data familiarisation.

**Step 2:** Data were reduced through data coding that was relevant to the interview guide questions and addressed the research question.

**Step 3:** Each predetermined theme was populated with the relevant data.

**Step 4:** Each theme was then reviewed to ensure that it was populated with relevant data and that themes produced meaning with their associated data.

**Step 5:** Themes were then refined in relation to each other and the main theme of the social burden of cancer care in families.

**Step 6:** The write-up and reporting of findings were the final step of the thematic analysis, which culminated into this manuscript.

The socio-demographic information was organised into a table, with the use of pseudonyms to protect the participants’ identities and maintain anonymity.^28^ The analysis was conducted by the lead investigator under the supervision of the co-authors who were the overseers of the entire study. This formed part of the investigator triangulation that was used to ensure the dependability of the analysis process.^22^ Reflexivity was maintained throughout this process through constant consultation with supervisors and research peers.

### Ethical considerations

The study was approved by the Biomedical Research Ethics Committee of the University of KwaZulu-Natal (BE532/18). The ethics committee approved written informed consent, which was obtained from all the participants prior to participating in the study.

## Results

Table 1 illustrates the general characteristics of the FCs who participated in this study. The participants’ ages ranged from 21 to 84 years, with 14 and six participants being females and males, respectively. Family members cared for included parents, spouses, children, grandparents, grandchildren and aunts. Few (*n* = 3) of the participants’ family members had passed on because of cancer at the time of the interview.

**TABLE 1:** Family caregiver demographics and characteristics.

Participant pseudonym	Age	Gender	Patient’s relationship to caregiver	Type of cancer reported	Patient alive at time of FC interview?
Thembi	48	Female	Grandmother	Stomach cancer	No
Candice	21	Female	Aunt	Kaposi sarcoma	Yes
Mandy	82	Female	Grandson	Nasal cancer	Yes
Zandi	27	Female	Aunt	Lung cancer	No
Olivia	59	Female	Grandmother	Leukaemia	Yes
Ingrid	73	Female	Husband	Prostate cancer	Yes
Wendy	61	Female	Grandson	Leukaemia	Yes
Sanele	75	Male	Wife	Uterine cancer	Yes
John	84	Male	Wife	Uterine cancer	Yes
Andile	60	Male	Wife	Breast cancer	Yes
Max	63	Male	Mother	Breast cancer	Yes
Lucia	32	Female	Mother	Breast cancer	Yes
Tracey	73	Female	Daughter	Colon cancer	No
Cindy	61	Female	Husband	Prostate cancer	Yes
Steve	42	Male	Father	Oesophageal cancer	Yes
Linda	72	Female	Husband	Lung cancer	Yes
Mary	37	Female	Mother	Squamous cell cancer (carcinoma)	Yes
Dora	34	Female	Aunt	Breast cancer	Yes
David	67	Male	Wife	Breast cancer	Yes
Lauren	30	Female	Mother	Head and neck cancer	Yes

FC, family caregiver.

## Themes

There were two main themes along with their respective sub-themes culminating from the data analysis. These themes were (1) *dynamics of a cancer diagnosis*: changes in relationships, changes in social life and (2) *psychosocial impact of a cancer diagnosis*: social support systems, three key pillars of support through the lenses of FCs.

### Dynamics of a cancer diagnosis

#### Changes in relationships

The participants were asked about the relationship between themselves and the family member that they care for, given that the relationship had shifted from that of being simple family to that of FCs and patients. This was in terms of whether they got along or if the cancer diagnosis had affected their relationship in any way. The dominant view was that of a very close relationship with the patient, with minor and resolvable episodes of a soured relationship. There were also reports of the cancer negatively affecting the patients’ personality, but being unable to impact on their relationship, because of the caregiver’s and patient’s understanding of the situation presented by the disease. Participants also shared about the importance of family responsibility and the fulfilment of expected duties in the family setting. This was shown as one of the positive experiences of cancer caregiving as it cultivated stronger familial bonds.

‘We are very close, absolutely close up until now, with all that scolding and shouting and the rude counselling, he still looks at me and says I love you and I think for me that’s most important … It hasn’t been easy because I think with the illness and his mood swings, it’s not easy but we are understanding the pain he is going through.’ (Cindy, 61 years old, female)

**Strengthened relationships:** One participant reported to always have a good relationship with the patient, with the bond growing even stronger during the role changes. The caring had been reciprocated because they both had been sick and cared for each other. However, there was a lone voice asserting that she did not have a good relationship with the patient prior to being diagnosed with cancer, a phenomenon that changed when the cancer diagnosis occurred, culminating in a strong bond being established during the journey of caring. This was a rare and positive insight to the caregiving experience:

‘[*A*]s I said to you we were not close, we were never close, we became closer through her illness because she realised that I’m the one she’s forced to count on.’ (Thembi, 48 years old, female)

**Diminished relationship:** The opposite occurred for another participant, whereby the cancer diagnosis turned the good relationship to a bad one because of the cancer and the challenges that came with it. These challenges centred around the financial burden caused by the expenses brought about by the cancer diagnosis:

‘[*W*]e were very close before she got sick … now it’s not that great and mostly it is because of money, nothing else but money.’ (Andile, 60 years old, male)

**Inverted relationship:** Another participant alluded to one of the challenging aspects of caregiving that highlighted the difficulty of adapting to a new role of being a caregiver. This participant reported a drastic change to her life in the form of reversed roles where she was once dependant on her mother who was supportive on many levels and assisted with domestic duties. After the diagnosis, the mother could no longer be as active and helpful and was now dependant on the participant who found it difficult to adapt to this shift in roles:

‘[*M*]y life changed a lot, not so much my sisters, but my life changed a lot because I was the closest to my mother. Although I’m so big, my mother used to make my lunch for work, my mother will cook for me, I’ll come home, and I will eat her food. I will go to [*work*] and she would make my lunch, when I’m at work she would phone me, she would even do my washing like you know if I’m too busy working, so you can imagine my life was affected very badly.’ (Lauren, 30 years old, female)

**Family responsibility:** There were participants who viewed caregiving as part of their family responsibility to care for their loved ones. They did not see the caregiving role as a problem because of the value they placed on caring for their family member.

‘I wouldn’t say my life changed but there were certain things that I had to cut down to be with her … you know it’s not something that was so important, this [*caring for the family member*] was more important.’ (David, 67 years old, male)

‘I’m her spouse and I take full responsibility to anything that she needs, anything.’ (David, 67 years old, male)

One participant presented a more accepting approach to caregiving and described it as part of her spousal duty as a wife and as a way of good role modelling to her married daughters:

‘[*I*] don’t look at it that way, I take it as if I am doing my job and to also be a mentor to my daughters, I have daughters who are married and living in their own houses, so they should learn from me how things are done in a marriage.’ (Ingrid, 73 years old, female)

#### Changes in social life

Participants were asked to share how their caregiving experience had affected their lives in general and their social life in particular. A complete disruption was an overarching sentiment that participants used to describe the changes to their life as they knew it and a loss of a social life. This was particularly in reference to all their time and attention being redirected to the patient, which was reported as a challenging experience. Many participants reported that they had to cut down on their social activities and things they enjoyed most, because of caregiving responsibilities and not wanting to leave the patient unattended.

‘Couldn’t even eat. I couldn’t go out with my friends yoh [*giggles*] there is a lot of things that I could not do at that time, it was difficult, there was no fun, I would ask myself, why is it me that has to care for these people, is there no one else?’ (Zandi, 27 years old, female)

**Psychological distress:** A caregiver, who had lost hope, shared about the dramatic change in her life after the diagnosis, as she did not know much about cancer and the life expectancy of a cancer patient. This caused her psychological distress because of the uncertainty of her loved one’s future, which also disrupted her social life.

‘It [*life*] changed drastically, it changed a lot because I didn’t know if this cancer can be treated, I just lost hope and didn’t know how long I have with him … I can’t even leave this house any more … because I have to constantly check on him. I lose my mind as he was the one I could hold on to, even his father hardly comes around. It’s just him and I in this house [*cries*] and he is very sick.’ (Mandy, 82 years old, female)

Some participants reported a lack of sleep and how caregiving has affected their sleeping patterns as they must continuously wake up during the night to check up on the patient. They also have to wake up very early in the morning to check on the patient and prepare for the day ahead of which involved other daily activities in addition to caregiving. This results in both psychological and physical exhaustion:

‘It [*life*] has changed because I hardly sleep now, I just think that I can’t sleep I have to constantly check on him.’ (Steve, 42 years old, male)

**Spiritual growth:** For another participant, caregiving was not only seen as a complete disruption but also as an opportunity for deepening spirituality through difficult circumstances. This experience grew their spirituality in the family as they engaged in prayer more often which helped them deal with the cancer diagnosis. However, the cancer progressed rapidly, and they grappled with seeing the patient in so much of pain:

‘[*W*]e got more spiritual but, in the end, it got so bad we actually prayed the lord to take her … She was suffering, I said Lord why are you making her suffer, why don’t you just take her, take the pain. And I couldn’t handle it anymore.’ (Tracey, 73 years old, female)

### Psychosocial impact of a cancer diagnosis

#### Social support systems

Participants were asked about their support structure if they felt that they were supported by family, friends or neighbours. There were divergent opinions, whereby some participants reported having enough support, and others reported having no such support.

**Accessible social support:** Some participants sourced readily available social support from their immediate family members, friends and neighbours. Support from others was valued as it enhanced the caregiving experience. This shows the importance of availing a support system to caregivers:

‘[*I*] don’t have any support needs because the kids are around, and they are very supportive and helpful.’ (Andile, 60 years old, male)

‘Oh no I had good friends that supported me and my neighbour, she would sleep over, to give me a break … My family like I said, my son moved in with me to help me, gave up his place to be here to support me as he could and then if she got really sick … her friends pick up the phone, they come flying down here take her to hospital.’ (Tracey, 73 years old, female)

**Inaccessible social support:** One participant felt that she was on her own in the caregiving journey as her experience with the lack of support from others had prepared her to deal with difficult situations by herself. Although there was a lack of social support, she desired it to lessen the burdensome caregiving experience:

‘[*I*] think at the end of the day before illness you already realise that you know what, if anything would happen these people will not help, so when something did arise it was cancer, so you were already prepared that it was a problem on your shoulders.’ (Dora, 34 years old, female)

Contrary to the absolute absence of family support, one participant experienced family support that was limited to only financial support, relegating the physical and emotional support to one person who was the designated FC. The participant reported that her family always used work as an excuse to avoid assisting her with caregiving duties. She conceded that she expected more than just financial support from the rest of the family members. This spoke to the need for a family-centred caregiving approach rooted in collective participation by all family members and in all aspects of caregiving:

‘They [*rest of the family*] used to put in a lot of money but when it comes to helping physically with their hands they wouldn’t help, but money, financially she [*patient*] had everything that she needed and she always got it on time, even food if she wanted something today she would get it today on time but love, they [*rest of the family*] didn’t have it, they always had excuses about work … No one cared how I felt and how hard it was for me, it was better if someone would come home from work and ask me how it was today, was it difficult or not you know.’ (Zandi, 27 years old, female)

**Desired psychosocial and financial resources:** Participants shared about the type of support needs they expect as FCs in order to make their caregiving job easier and less burdensome. These needs included physical, emotional and financial support from other family members. The same needs were also expected from the healthcare system with added educational and training support in order for them to be able to provide better care to their family members. The above-mentioned support needs are evidence of the desired collaboration between FCs and healthcare system in patient care. Majority of the participants spoke about finances and how financial assistance can be useful in seeing to the patient’s caregiving needs. Education on cancer was also a support need that was mentioned by some participants in terms of training on how to care for a patient with cancer at home and to be educated about cancer as a disease. Transportation was also a support need for the participants, a provision of transportation for the patient to be taken to healthcare facilities and to collect medication when the need arose. Others needed to have a healthcare provider, such as a nurse, to do home visits to check on the patient and to see whether the FC is providing adequate care for the patient. These support needs can be addressed through a three-fold collaboration amongst the healthcare system, FCs and patients as previously mentioned:

‘[*I*] need financial support nothing else, because it’s our finances that get affected the most [*by cancer caregiving*].’ (John, 84 years old, male)

‘To get some sort of income, like when she [*patient*] needs to go to the hospital, I have to hire a car for her, because having to take public transport to town then to the hospital, is hard on her. So, I have to ask my neighbour and be able to pay him.’ (Max, 63 years old, male)

‘I need support maybe like for someone [*healthcare provider*] to come over especially during the day to check on him [*patient*] and how he is doing … just that it is important to teach people about cancer and how to care for a person who has cancer because when you are faced with this situation you are not aware what to do.’ (Steve, 42 years old, male)

‘Just for us to get medication when she [*patient*] needs it, to get someone who can assist us in that regard, who we can work together with to make it easier to get her medication. On days where the sun is too hot for her to go, maybe someone to assist with transport to the clinic and back because the clinic is far or someone to bring the medication to us here at home because when the sun is hot it becomes very difficult.’ (Candice, 21 years old, female)

#### Three key pillars of support and through the lenses of family caregivers

Participants were asked to mention the ways in which caregiving can be made easier for them and the actions that can be taken to make caregiving less difficult. This was with the intention of getting recommendations from the participants themselves on how to support FCs. Three main recommendations emerged, namely financial, psychosocial and educational support. There was a convergence of views in that main support should be provided by the government whilst expecting further auxiliary support from other family members, hence a need for a family-centred approach to caregiving.

**Financial support:** Most participants had recommendations for financial assistance, as the lack of finances was one of the main contributors to the increased burden of caregiving. The provision of transportation to healthcare facilities for the collection of medication or the delivery of medication to the patients was suggested as an intervention that may lighten the financial burden of transporting the patient to and from healthcare facilities:

‘I think the social worker department should have something, a caregivers’ grant or something you know, will be of some help.’ (Cindy, 61 years old, female)

‘I also think that we wouldn’t have been in this situation if you know like there was the government [*grant*] where you could say look this person’s got cancer can I get some money to sort them out.’ (Thembi, 48 years old, female)

‘My grandson has a father, but he has never helped with anything … not even once has he helped me, even when the child is critical in hospital, he has never come to see him … his grandpa, if I ask for help from his grandpa he always has to wait for pay day and when he helps financially, when I get my pension I have to pay him back.’ (Wendy, 61 years old, female)

‘The thing that will make it easy is getting her medication or someone to take us to the clinic and bring us back or someone to just bring the medication here to her … maybe it can be the people from the centre or the people from the clinic, the volunteers from there.’ (Candice, 21 years old, female)

One participant reported on how the transport provided by the hospital was helpful for her mother as her mother was once transported from one hospital to another for an operation and transported back home. The participant shared this as a recommendation of transportation for patients as it was an effective and helpful method:

‘[*W*]e were able to get all the help that we needed from the government hospital [*Greys hospital*], transporting her [*patient*] to Albert Luthuli hospital … The hospital [*Greys hospital*] provided everything for her to go there, everything for her to get operated, she stays there and she is okay until she comes back, even when she came back from Albert [*Inkosi Albert Luthuli Central Hospital*] they didn’t take her back to Greys [*hospital*]. They brought her back home, so everything was taken care of by the people from Greys [*hospital*].’ (Lucia, 32 years old, female)

**Psychosocial support:** Caregiver support groups and counselling were regarded an important intervention that could be beneficial to the psychosocial well-being of FCs and participants expected this support from the government.

‘Like I said, we need a counselling place where people can go with situations like this here and someone they can talk to, give them advice, do it like this, and do it like that, things like that … a support group, we can have it here.’ (Tracey, 73 years old, female)

‘Basically, the government should [*provide support*].’ (Mary, 37 years old, female)

**Educational support:** Training and knowledge transfer for FCs were also suggested by most participants, as an important intervention to be explored where FCs could be equipped with the essential skills to adequately care for their family members with cancer. The lack of knowledge about cancer also contributed to FCs’ fears of being inadequately equipped to provide proper care:

‘I would have loved to get maybe some sort of training on how to look after a person who has cancer and how to handle things if you find yourself in such a situation … because it was very difficult, so if I had someone close by to show me how to care for her even if it’s just to tell me what to do from a far you know.’ (Max, 63 years old, male)

‘I do think that there, maybe if like once you know that the person is ill, information like what the workshop does, they do that like you know when you become a foster parent at the Durban child welfare, they call you in and you go for classes about foster parenting, they should do something similar with cancer, explain to you because we want to know.’ (Thembi, 48 years old, female)

‘Just that it is important to teach people about cancer and how to care for a person who has cancer because when you are faced with this situation you are not aware what to do. Even at schools they should teach the learners on how to handle such situations when they have a sick person at home that needs caring.’ (Steve, 42 years old, male)

**FIGURE 1:**
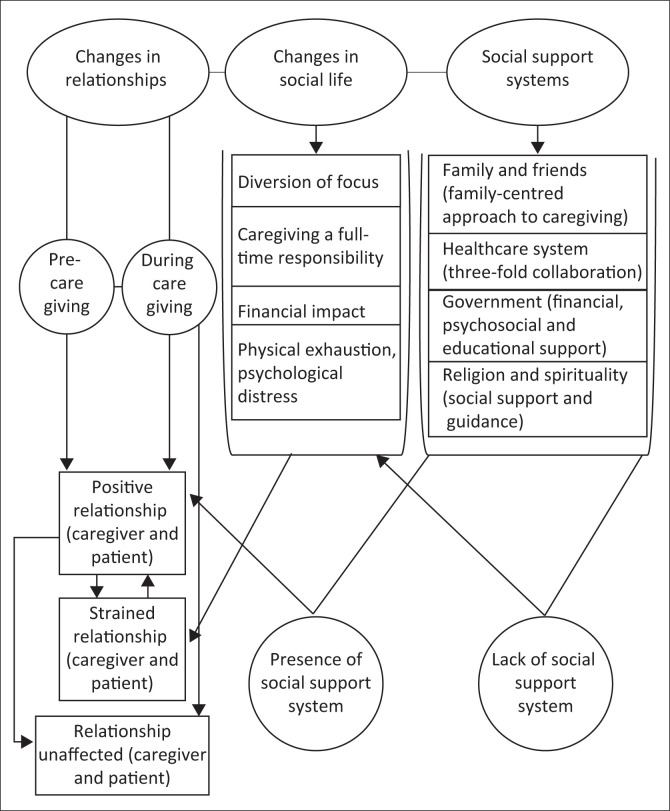
Conceptual framework on the dynamics and psychosocial impact of a cancer diagnosis for family caregivers.

Figure 1 presents an illustrative diagram that emanated from the results of this study and demonstrates the social burden and experiences endured by FCs during caregiving as a result of a cancer diagnosis in the family. In this framework, it is evident that, whilst caregiving is socially burdening, it can also yield positive relational outcomes. This is because of the increased time spent together in the FC and patient relationship, which is attributable to caregiving being a full-time responsibility. However, cancer caregiving creates social disruptions for FCs, which are exacerbated by the lack of a social support system. Nevertheless, the presence of an active social support system can potentially mitigate social disruptions and lighten the burden of caregiving.

## Discussion

A cancer diagnosis may change a person or negatively impact the personality of the person diagnosed as confirmed by this study. As mentioned by the participants in this study, as the patient becomes short-tempered and stubborn, the FC must then establish a sense of understanding of the patient’s situation to maintain a good relationship with the patient whilst the FC’s feelings remain unattended.^7^ This then confirms the findings of Girgis et al. that a cancer diagnosis has a greater impact on the family members than it does on patients as the family has to learn how to deal with the effect of a sudden cancer diagnosis and change in personality of the ill family member.^3^ This change in personality can affect the relationship between the patient and the FC as was found in this study. However, this change may not always yield negative results, as strengthened relationships between the FC and patient, post the diagnosis with cancer, were also reported in this study.

As stated by Maree et al., caregivers feel that they need to possess strength, not only for themselves but also for the patient, as well as the rest of the family, as re-affirmed by this study.^1^ Whilst this can be emotionally draining for the FCs, it can also increase their emotional capacity and resilience, as these virtues are often acquired through hardships.^29,30^ Although emotional capacity and resilience may be positive attributes, they are developed through experience and are helpful in coping with future similar difficulties, and thus, access to psychosocial support is vital in coping with current adversities.^29,31^ Although strengthened relationships were part of the outcomes of caregiving in this study, it must be noted that a cancer diagnosis can also bear detrimental outcomes to family relationships, arising from other challenges related to the cancer diagnosis, for example, an increase in medical expenses, as was found in this study. Therefore, lack of emotional, social and economic preparedness for an illness and difficulties in managing these challenges can be a cause of conflict within families.^3^

As reported in this study, a cancer diagnosis has proven to be a disruption to the lives of FCs. This is consistent with the findings of Girgis et al. that the role of caregiving disrupts the social lives of FCs.^3^ The participants in this study experienced a loss of social life as all their time and energy had to be redirected to caring for the patient. Family caregivers had to cut down on their social activities to pursue caregiving responsibilities. This study has shown that caregiving responsibilities also disrupt the sleep patterns of FCs, as they must constantly wake up during the night to check on the patient and wake up early in the morning to resume their caregiving roles. This is whilst also managing the general household duties, as caregiving is not limited only to caring for the patient. The findings of this study suggest that the lack of social support from family and friends would inadvertently increase the caregiving burden faced by FCs. This was confirmed by some participants in this study as they reported to have felt alone and that nobody cared enough to offer any kind of support.

Contrary to the above experience of some FCs, other participants did not view caregiving as a disruption to life but rather as a minor adjustment to their usual life, and that caring for the ill family member was the most important part of their social responsibility within the family system. Congruent with published evidence, this study found that through the caregiving process, a stronger bond and a sense of connectedness are established between the FC and patient.^6^

Research by Palos et al. revealed that, as the patient’s cancer progressed in severity, the caregiver gets exposed to greater sleep disturbances, exhaustion and stress.^32^ These findings were supported by this study, thereby confirming that caregiving also has a negative effect on the physical and psychological well-being of FCs. Spirituality also played a role in the caregiving process, as some participants reported to have an increased spirituality, which in turn enhanced their coping mechanism, this is also supported by other studies.^1,33^ Similar to the findings of Oyegbile et al., one participant in this study felt that caregiving was part of her spousal responsibilities, as a wife, upholding her marriage vows and commitment to her ill husband and being a good role model to her married daughters.^34^ The notion of spousal responsibility was not only one directional (wife to husband); hence a male participant in this study also held the same view that caring for his wife was his responsibility as the spouse. Acceptance of the caregiving role allowed these participants to embrace their roles as FCs; hence, they did not see it as having a negative impact on their lives.^34^ However, acceptance of the caregiving role does not nullify the difficulties that are faced by FCs and the social burden that is created by cancer caregiving.

Social support from family and friends has shown to have a positive impact on FCs. This was seen in this study where participants who reported to have enough social support from family and friends reported less caregiver burden. Butow et al. also reported similar findings, which revealed that social support from others was associated with healthier well-being of caregivers.^8^ Participants in the study by Maree et al. reported that they experienced a lack of support from the rest of their family members.^1^ The same was found in this study where some participants expressed their disappointment in the lack of support from other family members. This suggests that social support, especially from family members, is desired by FCs and that it can lighten the caregiving burden and improve the well-being of FCs as suggested by Nissen et al.^13^

This study suggests that a supportive family structure is likely to yield a positive impact on the FCs social well-being and alleviate the caregiver burden by working together as a family in all aspects of caregiving. Caregiving duties should ideally be shared amongst the family, thereby giving the primary FC a break, as suggested in this study, whereby one participant expressed her frustration about the lack of physical and emotional support from her family, despite assisting financially. This suggests that a supportive family structure is not only limited to financial matters although it is an important aspect of social support, FCs also yearn for emotional support. This may be difficult for small families, that is, a household of two people, one patient and one family member to take on caregiving duties. However, this is where the three-part collaboration amongst the patient, healthcare provider and the FC comes into play as suggested by Baas.^12^ The establishment of a partnership amongst the three parties sufficiently equipped with information and experiences shared continuously can be beneficial to the FCs. Such a partnership can also create a platform for FCs to voice their experiences and difficulties in caregiving. This approach can then be used to formulate a support system for families and FCs within the healthcare system and can be helpful to FCs of small families who are alone in caregiving. Furthermore, interventions, such as support groups for FCs, caregiving educational classes, counselling sessions for both FCs and patients, having a healthcare provider do home visits to check on the patient and assist the FC with caregiving duties and even financial support, may decrease some of the challenges and the unpreparedness of caregiving faced by the FCs, as suggested by the participants in this study. This study has revealed that the recognition of FCs and their needs, and the provision of social support from the healthcare system can positively impact the lives of FCs and improve their psychological, financial and social well-being.

The results of this study have affirmed that FCs suffer from many unmet needs, which, in turn, contribute to the FCs’ daily caregiving burden. These unmet needs include financial, psychosocial, educational and transportation needs, assistance from healthcare providers and emotional support from family and friends. It can be said that support from the rest of the family can lessen the caregiving burden as one participant reported to not have any support needs as he had enough support and help from his children. The provision of support was expected from the government in terms of financing caregiving support needs as many participants needed financial support in their caregiving role. Some participants expected their support needs to be provided for by their families, which they felt was lacking and thus, adding to the caregiving burden as they found themselves alone in caregiving.

This study showed that FCs need holistic interventions that will assist in lightening the caregiving burden. Many participants made recommendations that were in line with their support needs, such as financial assistance in the form of a monthly FC social grant that can assist the families with the additional expenses that are cancer caregiving related. Psychological counselling for caregivers to see to the psychological distress that is brought about by the cancer diagnosis as well as caregiver support groups were all mentioned as potentially helpful holistic interventions. As further analysis revealed a lack of knowledge about cancer and the caregiving thereof in some of the participants, many suggested cancer educational programmes for FCs, designed to bridge the knowledge gap and equip FCs with the necessary capacity to adequately care for their loved ones affected by cancer. The provision of free patient transportation to and from healthcare facilities was proposed, based on the transportation being one of the financially and logistically challenging aspects of caregiving. The delivery of medication to the patient was also suggested by one participant as it could reduce travel costs associated with patient transportation and further lessen the financial burden associated with cancer caregiving, as in many instances, the caregiver accompanies the patient and thus resulting in increased travel costs.

The findings of this study are congruent with those by other researchers, especially in advocating for a family-centred approach to improving the caregiving experience.^1,8,35^ This study has also highlighted the knowledge needs of FCs as many participants in this study reported that a working knowledge of cancer caregiving would be helpful in the caregiving journey. This was also highlighted by Given et al. that FCs require basic knowledge of the patient’s plan of care, and this can be achieved by regular interaction between the FCs and the healthcare providers.^36^ As participants mentioned psychological and educational support needs and recommended the provision of these needs, a psycho-educational intervention would lessen the caregiving burden as this intervention focuses on the provision of emotional and educational support to FCs.^36^ The introduction of non-medically trained lay healthcare navigators into the South African healthcare system may be useful as they provide guidance to patients and FCs.^37,38^ This guidance involves providing financial assistance, transportation, emotional support, explanation of treatment and coordination of care amongst other services within the healthcare system that are of benefit to the patients and FCs.^37,38^ The above recommendations can be helpful in the quest to alleviate the psychosocial and financial burden faced by FCs because of cancer caregiving.

## Strengths and limitations of the study

An important limitation to be noted is that data were generated from only one family member who was identified as the primary caregiver of the patient with cancer. Therefore, the findings may not be a complete representation of the whole family’s experiences. Despite the identified study limitation, this study is one of the few, if not the first, to be conducted in KZN, South Africa, on the social burden experienced by families caring for members living with cancer and has provided the participants with a platform to voice their challenges as FCs.

## Conclusion

The results of this study have re-affirmed that cancer caregiving is a difficult and time-consuming task for FCs. This study makes a case for a family-centred approach to caregiving and a partnership amongst patients, healthcare providers and FCs. There is a need for FCs to be recognised in the healthcare system with the development of policies and interventions that will support the needs of FCs and lighten the caregiver burden. This will further enable FCs to provide better care and support for their family members with cancer and thus, positively impact cancer survivorship.
